# Identifying Biomedical Entities for Datasets in Scientific Articles: 4-Step Cache-Augmented Generation Approach Using GPT-4o and PubTator 3.0

**DOI:** 10.2196/73822

**Published:** 2025-11-20

**Authors:** Claudia Giuliani, Gita Benadi, Felix Engel, Jonas Werner, Manuel Watter, Guido Schwarzer, Olaf Groß, Robert Zeiser, Harald Binder, Klaus Kaier

**Affiliations:** 1 Institute of Medical Biometry and Statistics, Medical Faculty and Medical Center, University of Freiburg, Stefan-Meier-Str. 26, Freiburg, 79104, Germany, 49 076127083739; 2 Institute of Neuropathology, Medical Faculty and Medical Center, University of Freiburg, Freiburg, Germany; 3 Center for Integrative Biological Signaling Studies, University of Freiburg, Freiburg, Germany; 4 Department of Medicine I, Medical Faculty and Medical Center, University of Freiburg, Freiburg, Germany

**Keywords:** metadata annotation, biomedical entities, GPT-4o, PubTator 3.0, cache-augmented generation, CAG, artificial intelligence, AI

## Abstract

**Background:**

The accurate extraction of biomedical entities in scientific articles is essential for effective metadata annotation of research datasets, ensuring data findability, accessibility, interoperability, and reusability in collaborative research.

**Objective:**

This study aimed to introduce a novel 4-step cache-augmented generation approach to identify biomedical entities for an automated metadata annotation of datasets, leveraging GPT-4o and PubTator 3.0.

**Methods:**

The method integrates four steps: (1) generation of candidate entities using GPT-4o, (2) validation via PubTator 3.0, (3) term extraction based on a metadata schema developed for the specific research area, and (4) a combined evaluation of PubTator-validated and schema-related terms. Applied to 23 articles published in the context of the Collaborative Research Center *OncoEscape*, the process was validated through supervised, face-to-face interviews with article authors, allowing an assessment of annotation precision using random-effects meta-analysis.

**Results:**

The approach yielded a mean of 19.6 schema-related and 6.7 PubTator-validated biomedical entities per article. Within the study’s specific context, the overall annotation precision was 98% (95% CI 94%-100%), with most prediction errors concentrated in articles outside the primary basic research domain of the schema. In a subsample (n=20), available supplemental material was included in the prediction process, but it did not improve precision (98%, 95% CI 95%‐100%). Moreover, the mean number of schema-related entities was 20.1 (*P*=.56) and the mean number of PubTator-validated entities was 6.7 (*P*=.68); these values did not increase with the additional information provided in the supplement.

**Conclusions:**

This study highlights the potential of large language model–supported metadata annotation. The findings underscore the practical feasibility of full-text analysis and suggest its potential for integration into routine workflows for biomedical metadata generation.

## Introduction

Long-term university-based research institutions, such as the German Collaborative Research Centers (CRCs), which can be funded for up to 12 years, require controlled and structured sharing of data and documents, with accurate, interoperable metadata being crucial for long-term data reusability and understanding [1-6] in accordance with the findability, accessibility, interoperability, and reusability (FAIR) principles [7]. To meet the requirements of the funding agency, the CRC *OncoEscape* (CRC 1479) adopted a dedicated metadata schema for describing its datasets [8]. These metadata are entered and stored in a central system, the *fredato* [9] platform, which makes the research data findable for the entire consortium and other users of the system. Currently, scientists have to manually fill the metadata forms, which often leads to suboptimal compliance. We therefore investigated methods for introducing an automated yet human-validated process for enriching datasets with relevant metadata.

For biomedical research data, a particularly important type of metadata is biomedical entities, such as organisms, cell lines, or genes. If published research articles and the associated datasets are tagged with these biomedical entities, it is easier for scientists to find relevant datasets for their research and to reuse these data. As manual entity annotation is often a time-consuming task, it is desirable to automate this process as much as possible. One method of automatic biomedical entity identification is named entity recognition, which involves identifying spans of text that represent named entities (eg, “mouse”) and tagging them with the appropriate category (eg, “organism”) [10]. Entity recognition is challenging due to difficulties in segmentation, that is, determining whether (1) a term represents an existing biomedical entity and (2) whether it is appropriate for describing the datasets that are presented in the corresponding research article.

Large language models (LLMs) can be used for entity recognition, and they can significantly increase the speed of biomedical entity documentation [11]. While LLMs exhibit strong language capabilities, they are prone to generating incorrect information, often referred to as hallucinations [12-16]. A possible way of mitigating this problem is to restrict the terms identified by the LLM to a set of known true bio-entities. This restriction can either be directly specified in the prompt to the LLM, or applied retrospectively by validating the list of terms suggested by the model.

In this manuscript, we present a newly developed method for article-based metadata annotation prediction based on the use of LLMs and the cache-augmented generation (CAG) method [17]. In particular, we used the LLM GPT-4o (OpenAI) in combination with PubTator 3.0, a tool built by the National Library of Medicine to identify several entities in the literature using state-of-the-art artificial intelligence (AI) techniques [18,19], and a dedicated metadata schema developed to describe data collected for a specific research area [8]. With this 2-fold combination of validation and constraint of the LLM, we intended to provide a reliable and extended list of annotation suggestions, exploiting the strengths of both PubTator, such as comprehensive genes and chemicals lists, and the dedicated metadata schema, which includes relevant mouse and cell lines that are often created in-house. This multipronged approach directly mitigates the risk of LLM “hallucination” by grounding the model’s output in 2 sources of truth: the externally validated PubTator 3.0 database and the domain-specific, curated metadata schema. The approach consists of four steps: (1) GPT-4o–based full-text analysis and suggestion of biomedical terms, (2) PubTator-based validation of suggested terms from step 1, (3) full-text analysis restricted to schema-related terms, and (4) combination of schema-related and PubTator-validated terms.

Why use both PubTator and a schema? PubTator 3.0 provides high-precision normalization for universal entities (eg, genes and chemicals) via canonical IDs, whereas the consortium schema captures project-specific concepts (eg, in-house mouse and cell lines, endpoints, and sample processing). Combining them lets the LLM ground standardized entities and still surface domain-specific terms that PubTator does not index. In practice, PubTator reduces false positives from free-text generation, while the schema broadens relevant coverage within the consortium’s scope. The merging step yields a single suggestion list with clear provenance (PubTator-validated entities vs schema-constrained entities).

We applied this 4-step approach in the context of the CRC *OncoEscape* (CRC 1479) and tested the feasibility of the approach using a structured, paper-based, and supervised annotation double-checking process. This process involved face-to-face interviews with the scientists who authored the respective article and enabled us to calculate a first estimate of the proportion of correctly predicted annotations. In detail, we evaluated the feasibility and precision of a 4-step cache-augmented pipeline for article-based metadata annotation using GPT-4o, PubTator 3.0, and a domain schema. The primary end point was precision of suggested annotations, assessed by supervised author validation for 23 OncoEscape articles. Secondary endpoints included the numbers of schema-related and PubTator-validated entities per article and a subsample analysis with and without supplemental material.

*OncoEscape* is a CRC funded by the German Research Foundation. The scientific goal of the consortium is to study oncogene-driven immune escape from the perspectives of clinical and basic tumor biologists and immunologists. The research of the CRC builds on the hypothesis that oncogenic signaling and immune escape mechanisms are closely connected. This concept implies that targeting oncogene-driven immune evasion could transform treatments that are currently mainly palliative into potential cures for different types of cancer.

## Methods

### Study Design and Data Sources

We included a total of 23 articles that were published during the first funding period of *OncoEscape* (2021‐2025) and for which a scientist responsible for authoring the article had agreed to take part in face-to-face interviews (Table S1 in Multimedia Appendix 1). We deliberately implemented this restriction, as we assumed that the social control arising from the presence of a member of the research data management group would be most appropriate to obtain a reliable assessment of the precision of the 4-step approach. All participants in the face-to-face interviews carefully checked the annotation predictions. In a subsample (n=20), available supplemental material was also included in the prediction process and 2 prediction results were validated in the face-to-face interviews. Of the 3 remaining articles, 1 had no supplemental material, 1 had such a large supplemental material section that the LLM was not able to analyze it correctly, and 1 was not considered in the face-to-face interviews.

### LLM-Based Annotation Prediction

**Figure 1.**
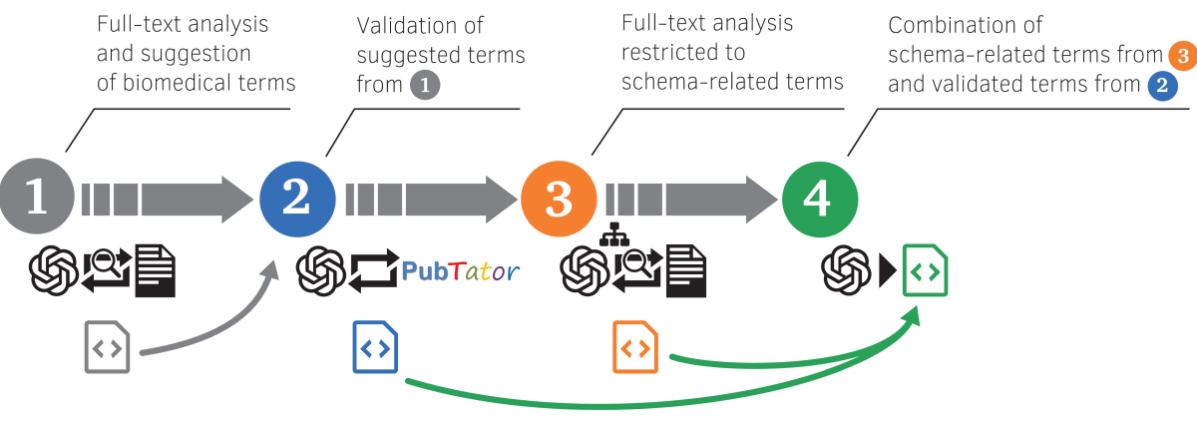
A 4-step cache-augmented generation approach for annotation prediction.

A systematic 4-step approach was used for annotation prediction (Figure 1): (1) ChatGPT (OpenAI) was initially tasked with generating relevant entities based on the full text of the paper; no further restrictions or suggestions were made, but the LLM was instructed to ignore the discussion and bibliography for its predictions. (2) The resulting entities were then validated using the PubTator 3.0 database. (3) Next, ChatGPT was instructed to reanalyze the full text of the manuscript but with the task of identifying entities defined in a predefined schema. For this purpose, a previously developed dedicated *OncoEscape* metadata schema [8] was included in this prompt in a tree-like structure. (4) Finally, a combined evaluation of the results from steps 2 and 3 was prompted, with the clear order of list only those PubTator-validated entities that had not already been identified through the schema extraction in step 3.

This process requires comprehensive textual analysis and the integration of knowledge, a method commonly referred to as CAG. In recent years, retrieval-augmented generation (RAG) has emerged as the standard approach for customizing LLMs to meet specific informational needs. However, with recent advancements in long-context LLMs, it is now feasible to eliminate the need for RAG by directly embedding all proprietary information within the prompt [17]. RAG’s reliance on real-time retrieval could introduce latency and potential errors in document selection, especially with large datasets. In contrast, CAG, by preloading all relevant resources into the LLM’s extended context, has been found to eliminate retrieval latency and minimize errors.

On the basis of the recommendations of the developers of PubTator 3.0 [19], we created a custom GPT with PubTator 3.0 augmentation following the instructions of the authors [20]. The above-described 4-step approach was operationalized using separate prompts in the chat interface from OpenAI, where the publication text was uploaded in the first step as a PDF file. Refer to the supplemental material for further details of the 4-step approach and specifically Table S2 in Multimedia Appendix 1 for the prompts used. All analyses were conducted using ChatGPT-4o-2024-11-20. Please note that this multistep prompting approach involved an iterative process in which ChatGPT connected to PubTator 3.0 to ensure reliability of the results. For each entity generated in step 1, step 2 instructed ChatGPT to query PubTator to retrieve a standardized entity ID, ensuring the validation of suggestions and alignment with recognized biomedical terms. Step 3 served the purpose of aligning and categorizing the biomedical entities suggestion with the dedicated *OncoEscape* metadata schema. This iterative process of generation, validation, and evidence gathering ensures that only scientifically grounded and relevant entities are carried forward and thereby overcomes some of the hallucination problems that arise when prompting ChatGPT in an unspecific way. Furthermore, the multistep prompting approach enables us to separate the capabilities of annotation prediction based on a schema and those of PubTator. In other words, the PubTator-based predictions can consider the scientific context from the paper and are not limited by the schema’s incompleteness. The PubTator-validated entities are either linked to a category but not included in the list of schema-defined entities, or they are entities that could not be associated with any OncoEscape schema category. In contrast, PubTator includes 6 categories of annotated entity types: gene, disease, chemical, variant, species, and cell line; the “FindEntityID” end point used in the GPT + PubTator implementation, however, does not consider species and cell line entries. This limitation is not relevant for the overall suggestion completeness because the *OncoEscape* metadata schema contains relevant species and dedicated cell lines used in the specific research area.

### Validation of LLM-Based Annotation Prediction

In this study, the proportion of correctly predicted annotations was chosen as the metric to measure the prediction capabilities of the LLMs. The proportion is computed for each paper and experimental setting (with or without supplement; total, schema-related, and PubTator-related suggestions). To identify correctly predicted annotations, we used a supervised annotation double-checking process. This process involved face-to-face interviews between a member of the research data management group and a senior scientist responsible for authoring the article. The research data management group member acted as a neutral facilitator and clarified ambiguities in the annotations without influencing the evaluation outcome. Scientists were instructed to consider the datasets referenced in the article as the context for evaluating annotations. Therefore, a standardized instruction sheet was used across all interviews to ensure consistency in interpretation. Each annotation suggested by the 4-step approach was examined to determine whether it accurately described the datasets used for the article. If an annotation was suspected to be incorrect, participants were encouraged to consult the methods or results sections of the article, which were provided in the paper for quick verification. Annotations deemed incorrect based on suspicion or evident errors could also be marked incorrect without comprehensive cross-checking to prioritize efficiency. The process was conducted in a supervised manner, and a 5-minute time span was recommended for a single article. This 5-minute face-to-face evaluation method ensured a practical balance between efficiency and reliability, enabling the rapid assessment of the 4-step approach.

### Statistical Analysis of the Interview Outcomes

To estimate the LLM prediction accuracy across all studies, methods for the meta‐analysis of single proportions were applied [21]. Due to the small number of entities per study, the Freeman-Tukey double arcsine transformation was used [22]. This transformation stabilizes the variances, makes the meta-analysis more robust when proportions are close to 1, and enables working with small sample sizes. A random-effects model using the restricted maximum likelihood estimator for between-study variance was applied. In addition, the behavior of the LLM, which is intrinsically stochastic, as well as the possible variability in the evaluation of LLM suggestions by different authors justified the calculation of CIs for the accuracy predictions in individual papers. Differences between the number of article-based and article + supplement–based biomedical entities were analyzed using the Wilcoxon signed-rank test.

In studies on natural language processing (NLP) using LLMs, false negatives are often also considered to determine the accuracy, as well as recall and the *F*_1_-score. In the present situation, where the goal of prediction was to maximize the number of correct annotations while also keeping the proportion of false-positive annotations as small as possible, it was not possible to compute the said metrics because the number of potential annotations per dataset had no clearly defined upper limit. Although it would be possible to ask the authors in the face-to-face interviews which biomedical entities they found missing, this question could not be addressed without additional effort. Moreover, we intentionally constrained the LLM by giving it information about the predefined CRC metadata schema so that the annotations were not only contextually appropriate to each paper but also relevant to the CRC’s specific scope.

### Ethical Considerations

This study’s primary data and analysis are based on nonhuman sources. The involvement of human participants was limited to informal expert consultations with professional colleagues to verify the study findings. No personal, private, or health-related data were collected from these individuals; the discussions focused exclusively on the interpretation of the nonhuman research data. According to national guidelines in Germany, particularly those established by the German Data Forum, research in the social and economic sciences that does not involve identifiable human material or susceptible participants may not automatically require formal review by an ethics committee. The German Data Forum’s guidance on research ethics emphasizes reflexive ethical conduct rather than a blanket ethics-votum for every empirical investigation [23]. Therefore, in line with these national standards and given that our study involves only voluntary expert interviews, no personal identifiable data collection, and no susceptible groups, we deemed a formal ethics committee approval unnecessary and instead opted for informed participation. These consultations were conducted in line with the principles of the Declaration of Helsinki and the guidelines for Good Research Practice of the German Research Foundation. All colleagues who participated did so voluntarily after being fully informed about the purpose of the discussion. Informed verbal consent was obtained from all colleagues who participated in the expert consultations.

## Results

**Figure 2.**
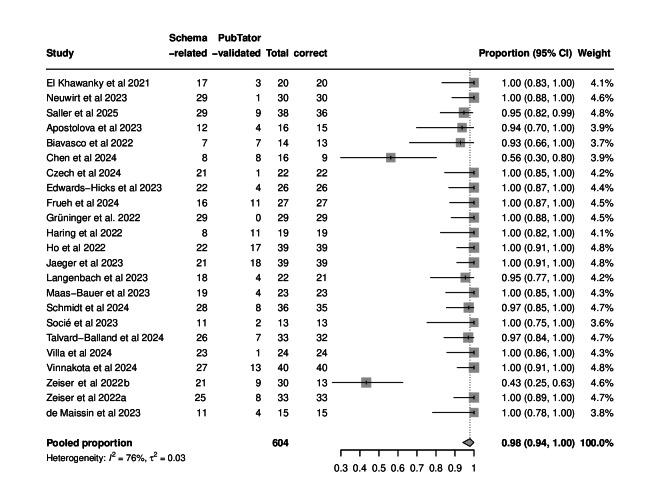
Precision of annotation predictions for each paper without supplement included in this study, along with pooled precision, heterogeneity, and τ^2^*.* Each dot and interval represents a paper-level precision estimate; the diamond indicates the pooled descriptive value [24-46].

A total of 604 biomedical entities were predicted for the 23 articles [24-46] (Figure 2). This equals a mean number of 26.3 biomedical entities per article, of which 19.6 were schema-related biomedical entities and another 6.7 were PubTator-validated biomedical entities. Overall precision, defined as the ratio of correct entities to the total number of annotation suggestions, was 98% (95% CI 94%‐100%), meaning that the vast majority of predicted biomedical entities were considered correct in the face-to-face interviews. An example of the LLM output for the article by Frueh et al [42] can be found in Table S3 in Multimedia Appendix 1.

Interestingly, the precision of schema-related biomedical entities was 97% (95% CI 91%‐100%), while the precision of PubTator-validated biomedical entities was 100% (Figures S1 and S2 in Multimedia Appendix 1). Most of the articles had a proportion of correctly chosen entities for the annotation close to or equal to 100% (Figure 2). Only 2 papers, namely, those by Chen et al [43] and Zeiser et al [44], had a markedly lower proportion of correctly suggested annotation entities, which contributed to the considerable between-study heterogeneity (*I*^2^=76%). Both these papers are clinical studies concerned with the transfer of basic research results into a clinical setting and had a number of false-positive basic research–related annotations that, although mentioned in the text, served as a rationale for the clinical approach rather than describing the underlying dataset. A post hoc sensitivity analysis excluding these 2 studies resulted in a pooled precision of 100% (95% CI 99%‐100%) with no substantial between-study heterogeneity (*I*^2^=0%).

In a subsample (n=20), available supplemental material was also included in the prediction process, and 2 prediction results, both those for the paper with and without supplement, were validated in the face-to-face interviews (Figure S3 in Multimedia Appendix 1). Interestingly, availability of supplemental material in the prediction process did not increase precision (98%, 95% CI 95%‐100%), with between-study heterogeneity of *I*^2^=67%. Moreover, the mean number of schema-related entities was 20.1 (*P*=.56) and the mean number of PubTator-validated entities was 6.7 (*P*=.68); these values did not increase with the additional information provided with the supplement and were compatible with the values obtained with the paper-only approach.

**Figure 3.**
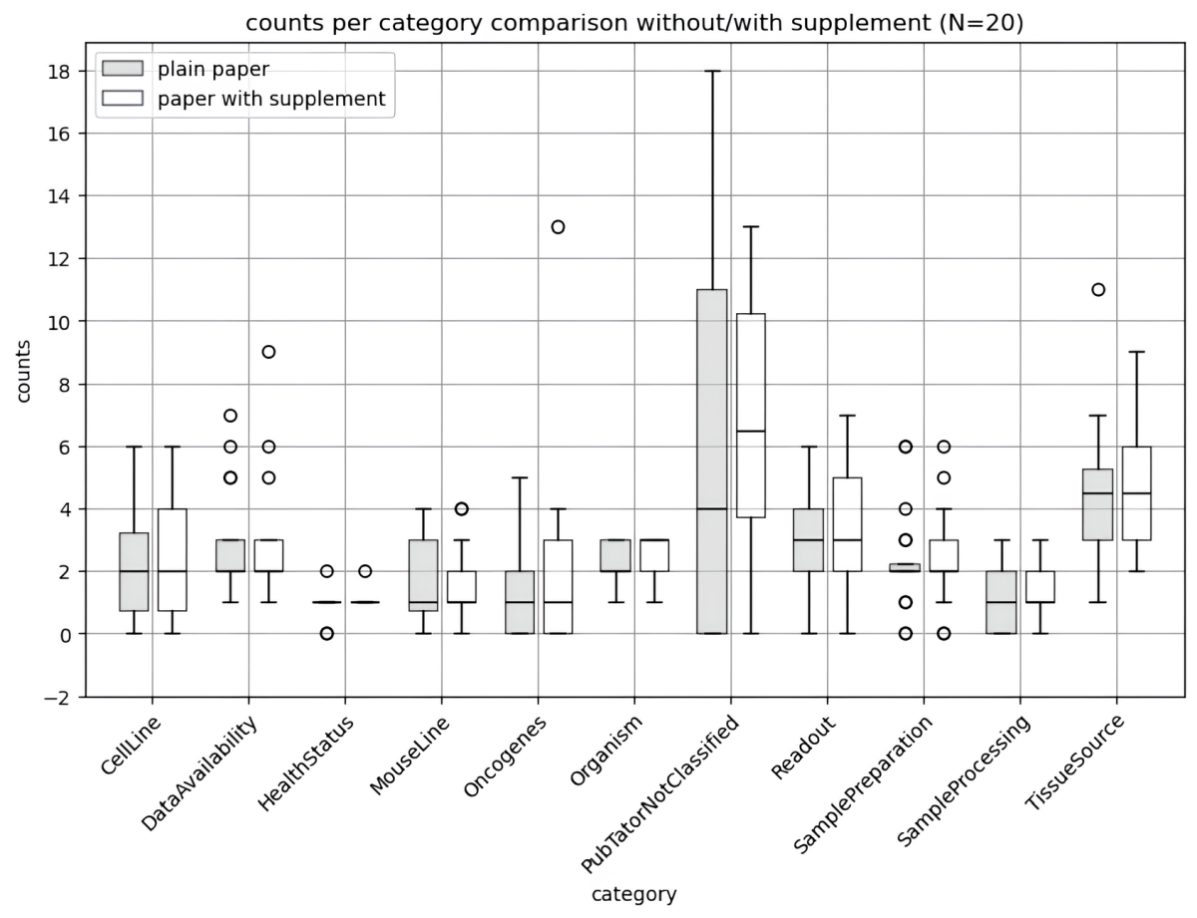
Counts of large language model annotation suggestions per category for papers with and without supplements. Only papers that included an annotated supplement are considered (n=20).

No statistically relevant differences were observed in the number of suggested entities for the papers without or with supplement, even when annotation suggestions were analyzed by category (Figure 3). Here, only the 20 papers that included annotation suggestions for the supplement are considered. The listed categories are as defined in the *OncoEscape* schema with 2 additional categories representing the availability of data: DataAvailability, which can be available publicly or on request as extracted from the paper, and PubTatorNotClassified, the PubTator-related entities that the LLM could not assign to a predefined category). Additional distributions are shown in the Multimedia Appendix.

The compatibility of the annotation suggestions obtained by analyzing the paper with or without the supplement was confirmed, both for the overall number of suggestions (*P*=.56) as well as for the suggestions divided per category. The detailed results of the Wilcoxon signed-rank tests are summarized in Table S4 in Multimedia Appendix 1.

## Discussion

### Principal Findings

Our 4-step approach involves a combination of GPT-4o, PubTator, and schema-related term analysis. This process requires comprehensive textual analysis and the integration of knowledge in an iterative process. We used CAG by preloading all relevant resources into the LLM’s extended context to accelerate information retrieval and minimize response errors. For instance, as both steps 1 and 3 involve full-text analysis, we accelerated step 3 by using CAG. Preloading the cache allowed the model to quickly access and process the entire article text, leading to faster suggestion of terms [17]. Moreover, CAG helped ensure that the model had a unified understanding of the entire article context, leading to improved consistency and accuracy in term prediction across all steps. This was crucial for maintaining the coherence and quality of metadata annotation throughout the process.

The high precision observed in our study underscores the potential of the CAG approach for streamlining metadata annotation. Moreover, because the supplemental material did not improve the average number of annotation suggestions or the proportion of correct ones and because it is complicated to retrieve the supplemental material in a standardized way, we concluded that the LLM annotation suggestions of papers without supplement should suffice in most cases. The reasons for this behavior are multiple: (1) supplemental material does not necessarily add methodological information and details. For example, in some cases, the supplemental material only includes tables and figures. Still, if we would use a deterministic approach, we would expect to obtain the same number of predictions each time and at least as many when the supplement is included. (2) LLMs are intrinsically stochastic, and therefore, the number of suggestions varies if the experiment is run multiple times. (3) Traffic on the server where the LLM runs and on the PubTator database can impact the number of suggested and PubTator-verified entities, as connection timeouts may occur under high load.

Although the abovementioned points 2 and 3 cannot be kept completely apart, we ran new experiments to show their combined effect on the number of suggested entities. For the paper by Saller et al [25] (without supplement), we ran the 4-step approach 10 times. On average, 46.7 entities were extracted, with a SD of 7.7. The minimum number of extracted entities was 37; the maximum number of extracted entities was 59. The count spread per category is shown in Figure S6 in Multimedia Appendix 1. The observed variability was not only in the number of extracted entities but also in the entities themselves. The total average Jaccard index over the 10 runs was 0.46. The summary Jaccard heat map for all combined categories is provided in Figure S7 in Multimedia Appendix 1. For selected categories, additional Jaccard heat maps and visualizations of the overlaps are shown in Figures S8-S11 in Multimedia Appendix 1.

The provided metadata schema had a nonnegligible impact on the quality and level of details of the biomedical entity suggestions and categorization by the LLM, especially for the categories not covered by the PubTator entities (eg, endpoint, sample preparation and processing, and tissue source). For example, if a dataset was processed with single-cell RNA sequencing but we gave the LLM only the first level of details of endpoint methods, it was able to provide only the given level of detail. In this case, “Sequencing” was selected as an annotation suggestion, which could be deemed wrong by the authors of the paper. In contrast, if we provided the LLM with additional endpoint levels of detail and, continuing with our example, we included different sequencing options, the LLM was able to identify that the data were processed with “single-cell RNA sequencing.” If the schema entities were passed to the LLM in a tree-like structure, the LLM was able to identify dependencies and reproduce the annotation depth. Likewise, the schema limited the capabilities of the LLM to correctly identify entities, especially if the paper in question did not analyze primary datasets in the context of basic research but was, for example, a clinical or modeling study. This was apparent in the lower proportion of correctly suggested entities in the papers discussing clinical studies. In both such articles, the basic research rationale was applied for the clinical approach, but the underlying datasets were of clinical nature.

The PubTator-validated entities identified outside the schema highlighted the potential of a hybrid approach that combines the structured annotation provided by the metadata schema with the flexibility to capture emerging concepts.

### Error Analysis

While the overall precision was very high at 98%, a detailed analysis of incorrect predictions was needed. The vast majority of errors originated from 2 specific articles (ie, those by Chen et al [43] and Zeiser et al [44]), which were clinical studies. In both cases, the LLM correctly identified biomedical entities mentioned in the text, but these entities were part of the introductory rationale or background discussion rather than describing the actual clinical datasets presented in the paper. For example, terms related to basic research (eg, specific mouse lines) were extracted because they were mentioned as foundational work, but they were irrelevant for annotating the human patient data that formed the core of the study. This highlighted a key challenge: the discrepancy between annotating an *article* and annotating the *underlying dataset*. Our instruction to the LLM to ignore the discussion and bibliography was an attempt to mitigate this, but it appeared insufficient for articles where the core methodology differed significantly from the background science discussed. This limitation was to some degree linked to our schema, which was heavily geared toward basic biomedical research and less suited for clinical or modeling studies. The lower precision in these cases underscored the need for schema adaptation when applying this method across different research domains. Overall, the limited generalizability of the current schema, which was deliberately designed for basic research datasets, explained why the approach underperformed in clinical or modeling contexts. From a responsible AI perspective, this underlined the need for domain-adaptive schema design, complemented by human-in-the-loop oversight to contextualize and correct outputs. Such an approach ensures accurate and context-appropriate annotations across diverse biomedical fields, aligning with the principle that automated annotation must always be supported by expert review.

### Comparison to Prior Work

In a recently published article, Alyafeai et al [47] compared a similar approach to metadata annotation based on papers on NLP datasets. Specifically, a 1-step approach was used to compare different LLMs. Compared to our approach, only step 3 was used. Within this step, all types of things were tested based on 52 manually annotated articles. Enabling web browsing led to a slight increase in accuracy, and the best results were achieved with the PDF format as input. An important difference from our approach was that the focus there was not on biomedical entities but rather on administrative aspects, such as license, language, domain, and other NLP-related aspects. Schilling-Wilhelmi et al [48] used a similar approach in the field of chemistry, but their results were only partially comparable due to the rapid development of LLMs (they used an early version of GPT-3.5). The same applies to the study by Singh et al [49], who were only able to achieve a precision of 0.46 based on a schema-based 1-step approach; they also used GPT-3.5. In contrast, Turner et al [50] used a more recent LLM (gpt-4o-2024-08-06) and yielded a high accuracy using a schema-based 1-step approach on papers from the field of psychiatry, with a much more promising accuracy (0.91-0.97). To our knowledge, no work has yet been done on a multistep approach with the aspect of validating biomedical entities. We did not perform head-to-head comparisons in this study; therefore, reported differences from prior work should be interpreted with caution, given variations in domain, model, and protocol.

### Limitations

Problems in extracting metadata annotation suggestions can arise when working on very long articles or extensive supplemental materials. This can happen when the available LLM context length is exhausted. Recent studies have shown that while LLMs benefit from larger context windows, their performance does not always scale linearly with increased input size, often exhibiting a “lost in the middle” effect, where information in the middle of long texts is processed less effectively [51]. Further studies on the effect of the LLM choice and its context length on the reliability of the annotation predictions are ongoing.

A major challenge of our approach was the distinction between article and datasets: the metadata annotation was focused on the respective underlying datasets; however, the prediction was conducted for each already published journal publication. This was intended, as we assumed that scientists can usually refer to the respective article when annotating their datasets with metadata. However, an article not only describes details of the respective datasets but also discusses aspects that go beyond them. We tried to solve this discrepancy by instructing the LLM to ignore the discussion and bibliography of the full texts during prediction (step 1). In view of some incorrect predictions, however, this may not have worked entirely. Nevertheless, we felt it was extremely important to further develop our 4-step approach on the full text of the article (instead of just feeding in parts of the article), as this seemed to be the only practical way to transfer it into a fully automated annotation process. The observed misclassifications underscore that responsible integration of LLM-based annotation requires a human-in-the-loop approach, ensuring that contextual background information is not inadvertently elevated to dataset-level descriptors. Furthermore, while our interview-based validation provided deep, expert-verified insights, we acknowledge that it may have introduced investigator bias and is less scalable than automated methods; this represents a trade-off between validation depth and breadth that future studies should address.

Our aim was to enrich the datasets collected within the research projects with metadata information to ultimately make them FAIR. To decrease the problem of having annotation predictions extracted from the analysis of the article and being unable to assign them reliably to a given dataset, we let the LLM retrieve information about available datasets after running the 4-step approach. For published datasets, the LLM was able to find the links to the datasets in the article and, when asked for it, could directly gather information about each published dataset through a web search. At least for published datasets, we can thus reliably assign our annotation suggestions to a specific dataset. However, some datasets were not published or only available on request. As in many cases, the datasets were not fully described; we could only state that data with certain properties and preparation were collected, but we could not have a one-to-one assignment of metadata to datasets. To improve the FAIRness of these datasets, additional infrastructural or institutional measures are needed, alongside further development of the LLM-based analysis methods. Additional measures could include the standardization of the dataset format with compulsory metadata information associated with it, interconnection of systems for automatic collection of metadata during the complete data lifecycle (data storage solutions, electronic lab notebooks, and data collection instruments such as microscopes), or establishing requirements for eventual publication of all datasets.

### Future Work and Recommendations

In general, LLM-based annotation enables the annotation of a large number of papers and datasets, thereby enhancing the FAIRness of the data. A comprehensive assessment not only of the quantitative but also of the qualitative advantages of the LLM automatic annotation compared with a diligent manual annotation is being investigated. It is already apparent that including the LLM-generated suggestions and validating them through PubTator is beneficial for having a comprehensive list of oncogenes and treatments considered in the experiments underlying the datasets. Future work should explore dynamic schema adaptation, for example, to include other areas of research such as computational modeling and integration of knowledge graphs [52,53] to represent complex relationships between biomedical entities, ultimately enhancing the comprehensiveness and accuracy of metadata annotation.

We showed that including supplemental material does not have a substantial impact on the number and quality of annotation suggestions. This finding is an advantage for the future automation of the annotation task because format and location of supplemental material are not standardized, which makes it difficult to automatically include in the annotation process. Nevertheless, we plan to further investigate the impact of supplemental material for different publication categories.

In addition, further studies are needed to evaluate the reproducibility of results, given the stochastic nature of the LLM output and the variability of human responses in interviews. In particular, we plan to investigate the impact of the choice of LLM on the annotation suggestions, as well as the variation of suggestions (number and extracted entities) when running the 4-step approach multiple times on a single paper in both scenarios: with and without supplemental material.

Furthermore, exploring advanced frameworks, such as Agentic AI, could enable the creation of autonomous workflows that not only perform the 4-step extraction but also dynamically handle error checking, query external databases for resolving ambiguity, and adapt the process based on the article’s specific domain. At the time we conducted this study, we had the impression that GPT-4o is fully used with the 4-step approach. However, future LLMs will likely be able to incorporate a larger number of agentic steps, which would allow the approach shown here to include additional validation, thereby moving toward a more robust and even more automated annotation system.

Another key priority for future work is to validate this 4-step approach on a larger and more diverse corpus of articles, extending beyond the OncoEscape consortium. To achieve this, we will need to develop a more scalable validation methodology, transitioning from face-to-face interviews to a structured, web-based survey system for authors. This will not only allow for the inclusion of hundreds of articles but also enable benchmarking against datasets from different biomedical domains, thereby providing a more robust assessment of the method’s external validity and its potential for widespread adoption.

Finally, future research should explore systematic ways of embedding responsible AI principles into metadata annotation workflows, including structured human oversight, transparent error reporting, and context-sensitive schema adaptation. In particular, developing flexible, domain-adaptive schemas that can be dynamically aligned with diverse biomedical contexts, such as clinical and computational modeling studies, will enable automated annotation to scale responsibly, with human oversight serving as a safeguard against contextually inappropriate outputs.

### Conclusions

With our 4-step approach, we showed that it is possible to streamline the extraction of annotation suggestions to achieve a comprehensive metadata description of the datasets underlying a biomedical publication. The interplay of the LLM, biomedical entity validation through PubTator, and the predefined metadata schema provided a reliable set of annotation suggestions. Providing annotation suggestions that the scientists just need to confirm will decrease the time that researchers need to spend on dataset documentation without compromising the quality of metadata annotation. Overall, the application of an automated 4-step approach represents a promising step toward improving research data FAIRness, as it enables broader and higher-quality annotation coverage compared with current annotation practices.

## Supplementary material

10.2196/73822 Multimedia Appendix 1 Supplemental tables and figures.

## Abbreviations

AI: artificial intelligence
CAG: cache-augmented generation
CRC: Collaborative Research Center
FAIR: findability, accessibility, interoperability, and reusability
LLM: large language model
NLP: natural language processing
RAG: retrieval-augmented generation
