# Supplementary material for: Identifying Biomedical Entities for Datasets in Scientific Articles: 4-Step Cache-Augmented Generation Approach Using GPT-4o and PubTator 3.0
Source: JMIR Form Res. 2025 Nov 20;9:e73822. doi: 10.2196/73822 (PMC12633840; doi:10.2196/73822)
Supplement: Multimedia Appendix 1 [file formative-v9-e73822-s001.docx]

**Supplemental Material**

*Table S1: List of OncoEscape Papers, naming as in Paper, used for the evaluation of the 4-step cache-augmented generation approach using GPT-4o and Pubtator 3.0 and their PubMedIDs*

| **Name** | **PubMedID** |
| --- | --- |
| El Khawanky et al. 2021 | 34750374 |
| Neuwirt et al. 2023 | 36649377 |
| Saller et al. 2025 | 39571574 |
| Apostolova et al 2023 | 37539479 |
| Biavasco et al 2022 | 35768570 |
| Chen et al 2024 | 38844797 |
| Czech et al 2024 | 38381845 |
| Edwards-Hicks et al 2023 | 36732424 |
| Frueh et al 2024 | 38170159 |
| Grüninger et al. 2022 | 35794338 |
| Haring et al 2022 | 34407601 |
| Ho et al 2022 | 35853161 |
| Jaeger et al 2023 | 36696631 |
| Langenbach et al 2023 | 37071397 |
| Maas-Bauer et al 2023 | 38199985 |
| Schmidt et al 2024 | 38885318 |
| Socie et al 2023 | 36827620 |
| Talvard-Balland et al 2024 | 38916965 |
| Villa et al 2024 | 38200005 |
| Vinnakota et al 2024 | 38741011 |
| Zeiser Socie et al 2022 | 34971577 |
| Zeiser et al 2022 | 35081254 |
| de Maissin et al 2023 | 37439488 |

**Prompt Strategy**

The prompts used for the 4-step strategy are added below (Table S2).

For machine readability of the results and to collect information on the availability of the datasets, three additional queries were run: 1) Dataset availability (public or on request); 2) If public available datasets exist, information about them should be retrieved, if possible with the same categorisation as for the paper; 3) consolidated results, with data availability, in tabular form for machine readability and analysis of results.

*Table S2: Prompt used for the 4-steps approach.*

| **Step** | **Prompt** |
| --- | --- |
| **1** | **Identification of Biomedical Entities**  Analyze the uploaded manuscript to identify all biomedical entities mentioned. Exclude entities mentioned only in the discussion section or the bibliography. Provide a comprehensive list of these entities. Complete this step entirely before proceeding to the next step. |
| **2** | **PubTator Analysis**  For each biomedical entity identified in Step 1, determine whether it is listed in PubTator. Provide a list of these entities along with their existence status in PubTator (e.g., listed or not listed). Do not include examples or elaborate explanations. Complete this step entirely before proceeding to the next step. |
| **3** | **Manuscript Reanalysis for Specific Aspects**  Reanalyze the uploaded manuscript to identify the presence of specific aspects listed below under each subheading. Only include aspects explicitly mentioned in the manuscript, excluding mentions from the discussion section or bibliography. Maintain the formatting and organization provided below, and provide a comprehensive list for each subheading.  **Organism**   - Cell line - Human - Mouse   **List of Cell Lines**   - B16.F10 - B16.F10^luc/GFP^ - B16.F10^OVA^ - AT-3 ^OVA^ - RENCA - KP1.9 - 4434-BRAF^V600E^ - MC38 - MC38 ^OVA^ - MC38^ROR1+/GFP+/Luc+^ - RMB1 - C1498 - C1498^GFP+ luc+^ - Yumm1.7 - Yumm1.7 ^OVA^ - MODE-K - JIMT-1 breast cancer - 32D - Platinum E - WEHI-3B - WEHI-3B^Luc/GFP^ - WEHI-3B^CD155KO^ - MOLM-13 - BAF-3 - MV4-11 - MV4-11^Luc+^ - MV4-11^NT Luc+^ - MV4-11^JunD KO (G4) Luc+^ - MV4-11^cJun KO (G11) Luc+^ - MV4-11^JunD KO (G64) Luc+^ - MV4-11^cJun KO (G69) Luc+^ - MV4-11^AXL KO (G1) Luc+^ - OCI-AML2^GFP+Luc+^ - OCI-AML3 - OCI-AML3^GFP+Luc+^ - Kasumi-1 - THP-1 - HL-60 - SEM - E2a-PBX - A20^GFP+Luc+^ - OCI-AML3^hp53 1961 (p53 KD) YFP+ dsRed+^ - OCI-AML3^hp53 Renilla (p53 WT) YFP+ dsRed+^ - RP1199.6 - RP1199.1 - RP1201.1 - RP1209.1 - RP12086 - Panc^ROR1+/GFP+/Luc+^ - STC-1 - NB-4 - ML-2 - K562 - KG-1 - KG-1α - MUTZ-8 - BV2 - FL83B - HLE - Colo800 - murine embryonic fibroblasts - OV-90 - BxPC3 - NCI-H2405 - HEK293T - Steinberger NFAT-reporter - SMMC-7721 - HepG2   **Tissue Source**   - Adrenal gland - Blood   - Blood plasma   - Blood serum   - Whole blood - Bone marrow - Brain - Embryonal tissue - Heart - Intestine - Kidney - Liver - Lung - Lymph node - Nerve - Skin - Spleen - Thymus - Urine - Feces - Vascular system   **Health Status**   - Cancer   **Mouse Line**   - C57BL/6J (wildtype) - C57BL/6J (wildtype) - C57BL/6-Nrastm1Tyj/J X Vav-Cre - C57BL/6JCya-Tigitem1/Cya - Tigitfl/fl;CD4cre/+ - CD155^-/-^ mice - Tigitfl/fl;Zbtb46cre/+ - Gal9^-/-^ - Nlrp3^-/-^ - Pycard^-/-^ - Casp1^-/-^ - Nt5e/Cd73^-/-^ - Gsdmd^-/-^ - Il1r1^-/-^ - Cmtm6^-/-^ - Osm^-/-^ - Osmr^-/-^ - FLT3-ITD - Ddit3^-/-^ - Rag1^tm1Mom^ - Rag2^-/-^gc^-/-^ - Tet2-ko (B6(Cg)-Tet2tm1.2Rao/J - Apcflox/flox - Col7a1^fl/fl^ - Pycard ^fl/fl^ - Xbp1 ^fl/fl^ - Adora2 ^fl/fl^ - Dnmt3afl/+ - Osmr ^fl/fl^ - Il1r1 ^fl/fl^ - Trp53fl/fl - Trp53LSL-R175H - KrasLSL-G12D - Vhl ^fl/fl^ - Rb1 ^fl/fl^ - Tak1 ^fl/fl^ - Atf6 ^fl/fl^ - Ptpn11 ^D61Y/+^ - SCLtTA/TRE-Cre - SCL-Cre - Osterix-Cre - LepR-Cre - Prx1-Cre - CD4-Cre - CX3CR1 ERT2 Cre - HexbCreERT2:R26R^Confetti^ - Mrc1CreERT2:R26R^Confetti^ - Cxcr4CreERT2:R26R^Confetti^ - Ksp1.3-Cre^ERT2^ - Ksp1.3-Cre^ERT2^; Vhl ^fl/fl^; Trp53 ^fl/fl^; Rb1 ^fl/fl^ - Mx1 Cre - LysM Cre iDTR - Cd4 Cre ERT2 - Villin-CreERT2 - Rosa26::CreERT2 - Tet2 ^fl/fl^Mx1-Cre - Rosa26::Cre ERT2 Kras^G12V^ - Rosa26::Cre ERT2 Jak2-V617F FLEX/+ - nATF6liv - Cas9 - MMTV-PyMT - ERAI - OT-1/Rag2^-/-^   **Sample Preparation**   - Cultured cells   - Adipocyte   - Cardiomyocyte   - Dendritic cell   - Embryonic cardiomyocytes   - Endothelial cell   - Epithelial cell   - Fibroblast   - hiPSC-CM   - hiPSC-FB   - Immune cell   - Leukocytes   - Lymphocytes   - Macrophage   - Monocytes   - Natural killer cells   - Neuronal cell   - Neurones   - Neutrophils   - Oocyte   - Pericytes   - Platelet   - Smooth muscle cells   - T cells - Isolated cells   - Adipocyte   - Cardiomyocyte   - Dendritic cell   - Embryonic cardiomyocytes   - Endothelial cell   - Epithelial cell   - Fibroblast   - hiPSC-CM   - hiPSC-FB   - Immune cell   - Leukocytes   - Lymphocytes   - Macrophage   - Monocytes   - Natural killer cells   - Neuronal cell   - Neurones   - Neutrophils   - Oocyte   - Pericytes   - Platelet   - Smooth muscle cells   - T cells - Tissue chunk - Tissue section (thin) - Tissue slice - Whole organ   **Oncogenes**   - cKIT-D816 - KRAS-G12D - FLT3-ITD - NPM ALK - MLL-AF9 - IDH1 - VHL - ATF6 - BRAF-V600E - CTNNB1 - PTEN loss / KMT9A - BAP1 - KRAS - KMT2A - AKT1 - cMyc - NFE2   **Sample Processing**   - Cleared fixed tissue - Formaldehyde-fixed and paraffin-embedded (FFPE) - Formaldehyde fixation - High-pressure frozen - None (Physiological solution) - OCT embedded and frozen   **Readout**   - Biomechanics   - Cell stretching   - Nanoindentation   - Sarcomer Length   - Single cell stretching   - Tissue stretching   - Tissue stretching Myodish - Clinical Imaging   - Computed tomography scan   - Echocardiography   - Magnetic resonance imaging   - Positron emission tomography - Computational modeling - Electron microscopy - Electrophysiology (clinical)   - Patch Clamp - current clamp   - Patch Clamp - voltage clamp   - Sharp Electrode - Electrophysiology (lab) - Flow cytometry / FACS - Light microscopy   - Confocal   - Multiphoton   - Slidescanner   - Widefield - Molecular Biology   - Aptamer (Proteomics)   - Bulk chromatin accessibility   - DNA Methylation (array)   - Enzyme-linked immunosorbent assay (ELISA)   - Gene Expression (array)   - Genotyping array and imputation   - Proximity extension assay (PEA)   - Quantitative polymerase chain reaction (qPCR)   - Standard biochemical assay   - Westernblot - Optical mapping - Optoacoustics - Sequencing   - ATAC sequencing   - DNA methylation (WGBS)   - RNA sequencing   - Single Cell chromatin accessibility   - Single cell RNA seq   - Whole exome sequencing   - Whole genome sequencing - Mass Spectrometry   - Interaction proteomics   - Metabolomics   - Proteomics   Complete this step entirely before proceeding to the next step. |
| **4** | **Consolidation of Results**  Combine the results of Steps 2 and 3:   1. For each subheading, provide a comprehensive, comma-separated list of aspects identified in Step 3. 2. Include any biomedical entities identified in Step 2 that are listed in PubTator but were not included as part of the aspects from Step 3. |

**PubTator3.0 Application Link:**

<https://chatgpt.com/g/g-67516c0cff048191912549e36ee550eb-pubtatorsearch>

**Example of the results of the 4-step approach**

The consolidated final results of the 4-step strategy using the manuscript by Frueh et al 2024 without supplement.

*Table S3: consolidated final results for Frueh et al 2024 without supplement.*

**Biomedical Entities from Step 3 (Schema-related)**

### Organism

- Cell line, human, mouse.

### List of Cell Lines

- Luciferase+ CD4+ T cells, WEHI-231.

### Tissue Source

- Blood, bone marrow, colon (intestine), spleen.

### Health Status

- None.

### Mouse Line

- C57BL/6 mice, BALB/c mice.

### Sample Preparation

- Cultured cells, isolated cells.

### Oncogenes

- None.

### Sample Processing

- None.

### Readout

- Flow cytometry, light microscopy, RNA sequencing.

### **Biomedical Entities from Step 2 Not Included in Step 3 (PubTator-validated)**

1. **BATF** - Listed as "@GENE_BATF".
2. **BACH2** - Listed as "@GENE_BACH2".
3. **IL-6** - Listed as "@GENE_IL6".
4. **IL-2** - Listed as "@GENE_IL2".
5. **TNF** - Listed as "@GENE_TNF".
6. **IL-10** - Listed as "@GENE_IL10RA".
7. **FoxP3** - Listed as "@GENE_FOXP3".
8. **Helios** - Listed as "@GENE_IKZF2".
9. **NLRP3** - Listed as "@GENE_NLRP3".
10. **IL-17** - Listed as "@GENE_IL17A".
11. **Luciferase** - Listed as "@CHEMICAL_3_oxo_C6_AHL".

**Forest Plots – Proportions of correctly annotated entities**

*Figure S1: Precision of schema-related annotation predictions for each paper (without supplement) considered in the study together with pooled precision, heterogeneity and τ^2^.*


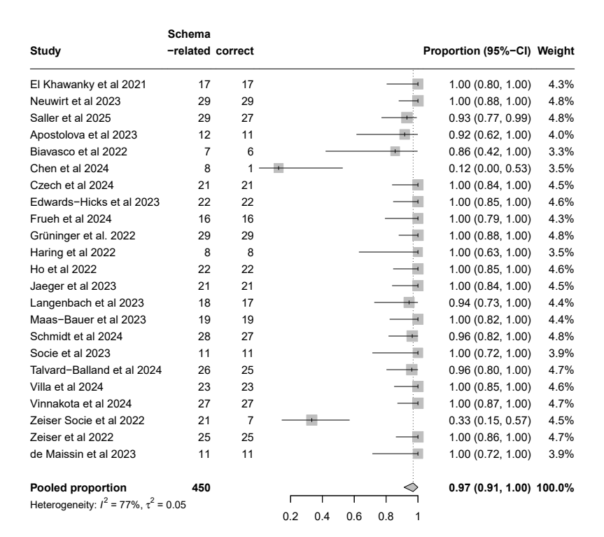


*Figure S2: Precision of PubTator-related annotation predictions for each paper (without supplement) considered in the study together with pooled precision, heterogeneity and τ^2^.*


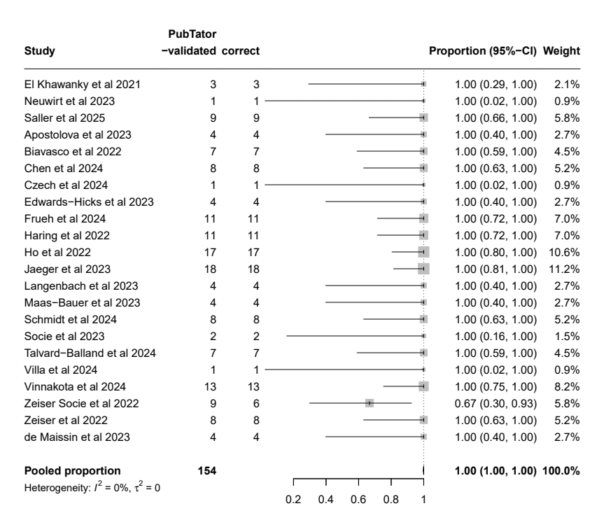


*Figure S3: Precision of PubTator-related annotation predictions for each paper with supplement (N=20) considered in the study together with pooled precision, heterogeneity and τ^2^.*


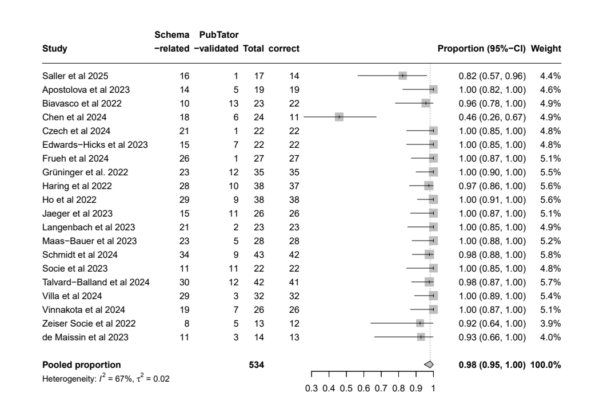


**Counts of annotation suggestions**

Additional distributions to show compatibility of number of annotation suggestions when the paper supplement is considered or not (Figure S4), as well as number of counts of annotation suggestions for the papers where no supplemental material was considered (Figure S5).

Exemplary for the paper Saller et al 2025 we run the 4-step approach 10 times to investigate the variability in number of suggestions and entities extracted from the publication.
Figure S6 and S7 show respectively the spread of number of annotation suggestions per category and the Jaccard heatmap for the 10 repetitions of the 4-step approach on the paper Saller et al 2025.

*Figure S4: Difference in counts of annotation suggestions for the papers for which the supplement was (1) not considered (2) considered (N=20).*


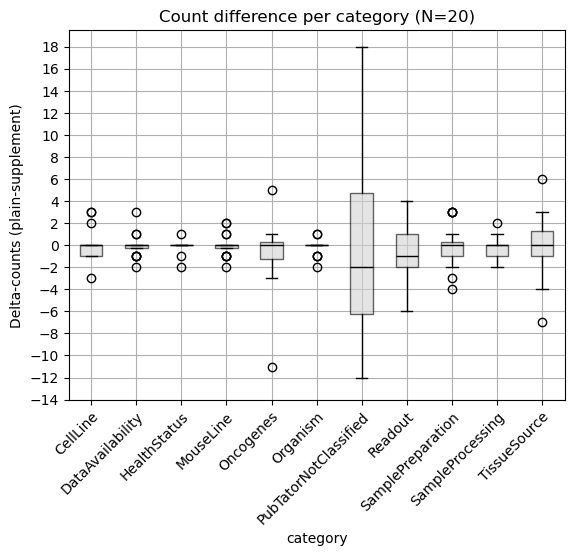


*Figure S5: Count spread of LLM annotation suggestions per category for papers with supplement not considered (N=23).*


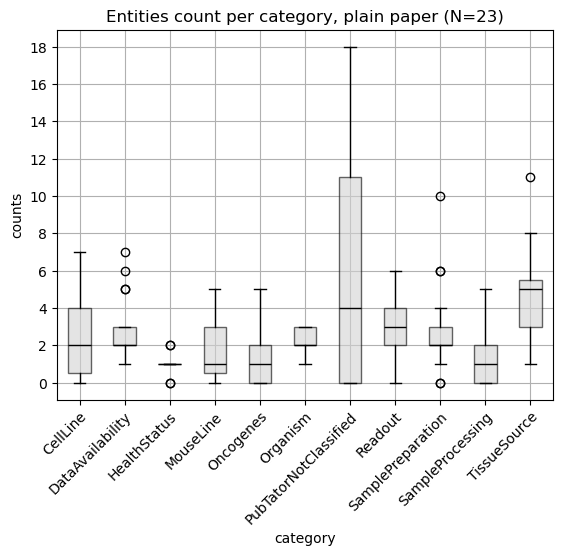


*Figure S6: Count spread of LLM annotation suggestions per category for paper Saller et al 2025. The 4-step approach was run N=10 times on the plain paper.*


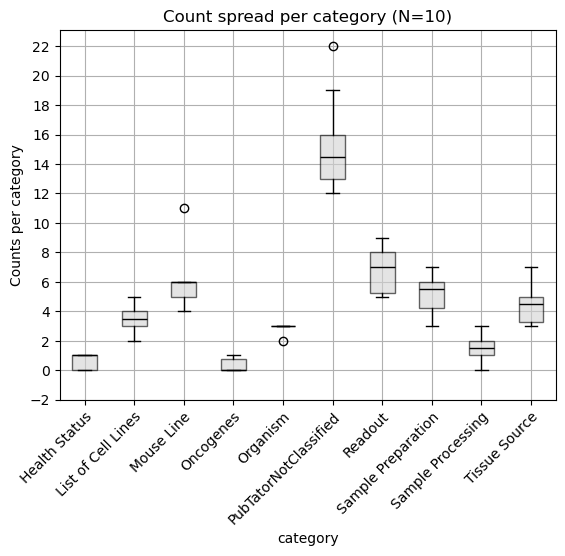


*Figure S7: Summary Jaccard heatmap for all categories combined for paper Saller et al 2025. The 4-step approach was run N=10 times on the plain paper.*
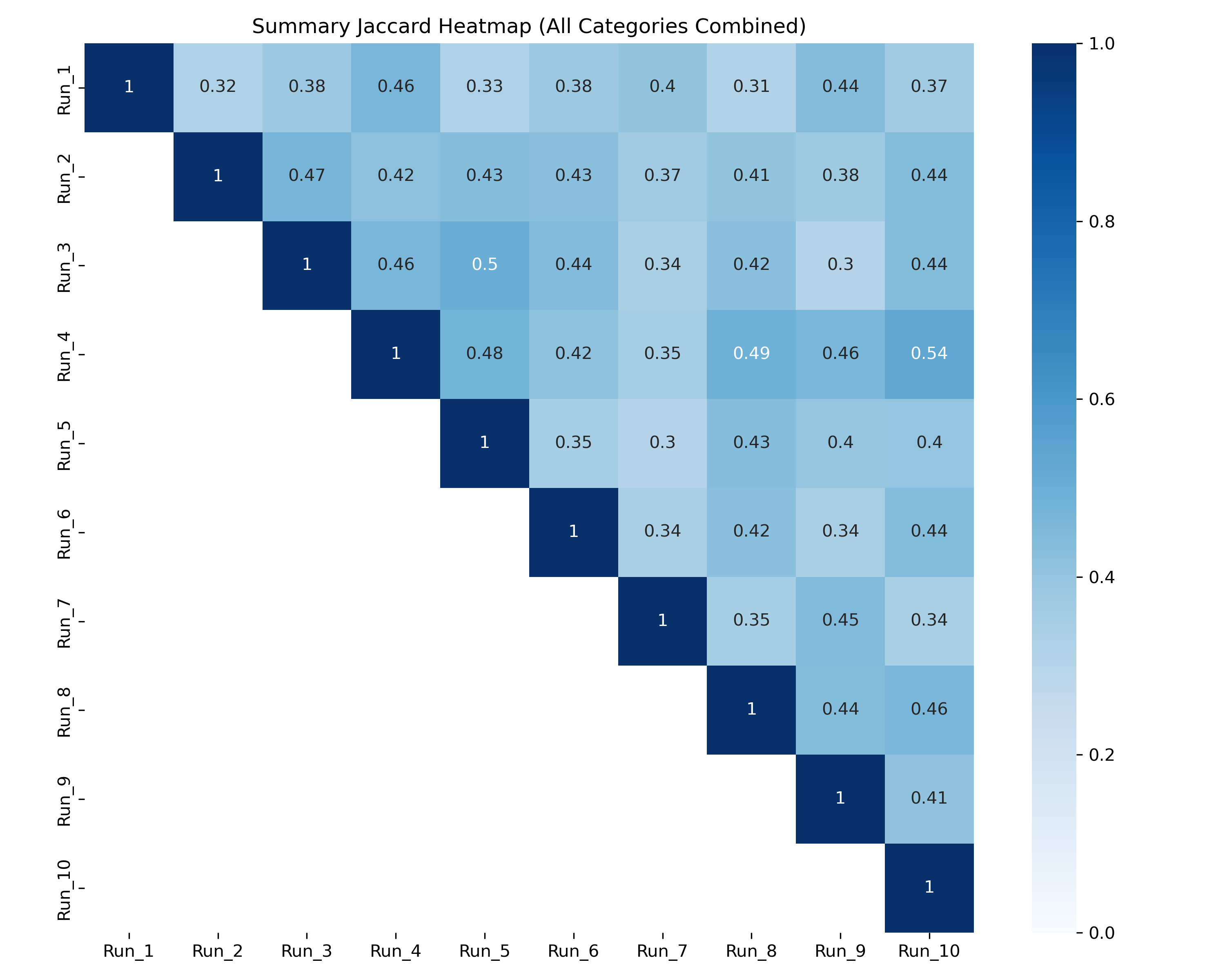


**
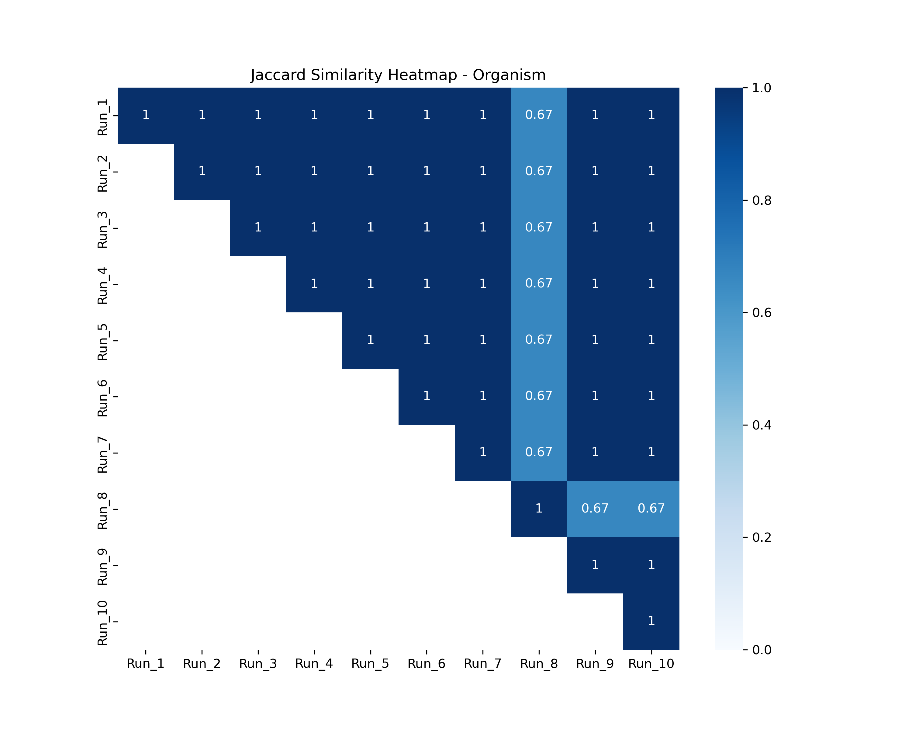

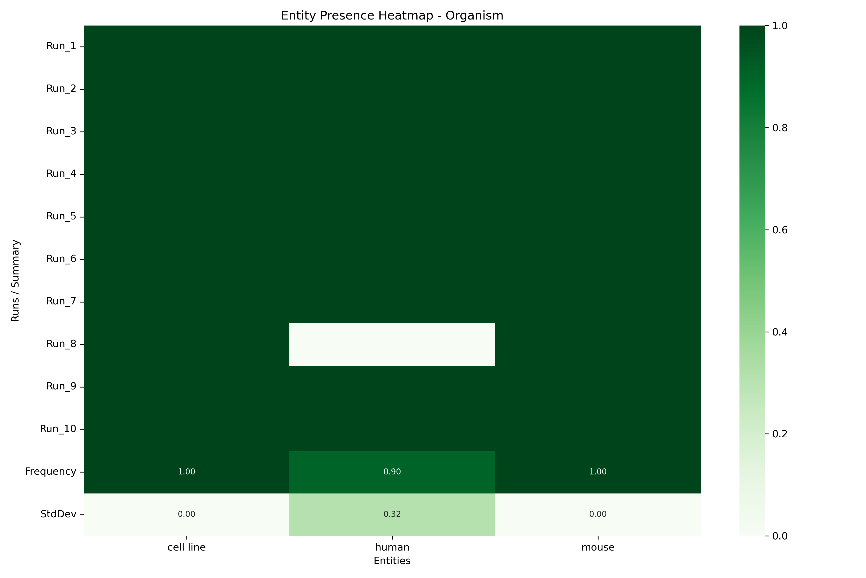
**In Figure S8 to S11 we show the Jaccard heatmap for three categories and the respective table of entities overlap over runs, the shown categories are organism, mouse line, tissue source and readout, respectively. A very high homogeneity is achieved for the category Organisms, a high overall Jaccard index was achieved also for the category Mouse Line, lower Jaccard similarities are observed for the categories Readout and especially Tissue Source.

*Figure S8: Summary Jaccard heatmap (left) and the entities overlap table for the category Organism (paper Saller et al 2025). The 4-step approach was run N=10 times on the plain paper.*

**
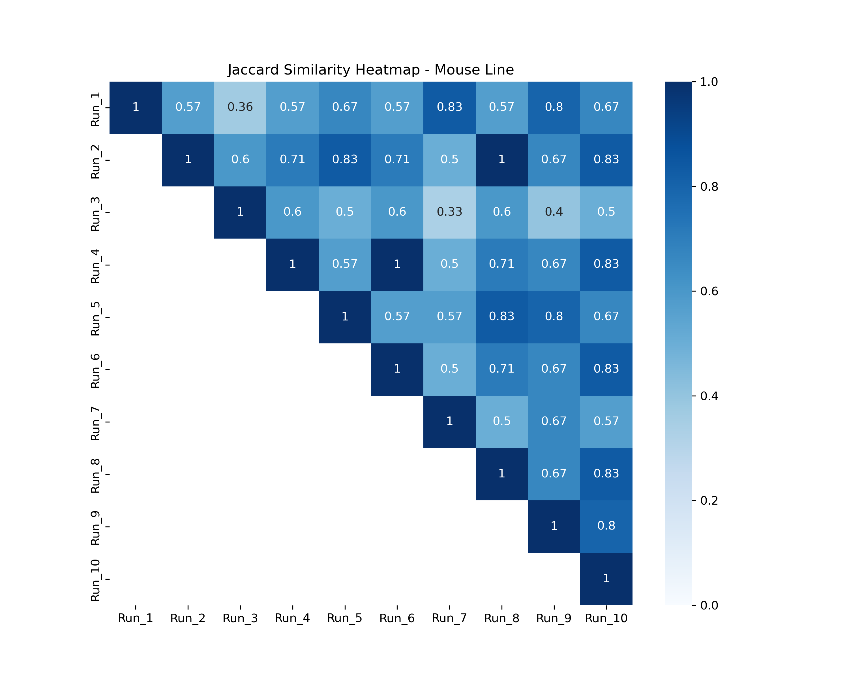

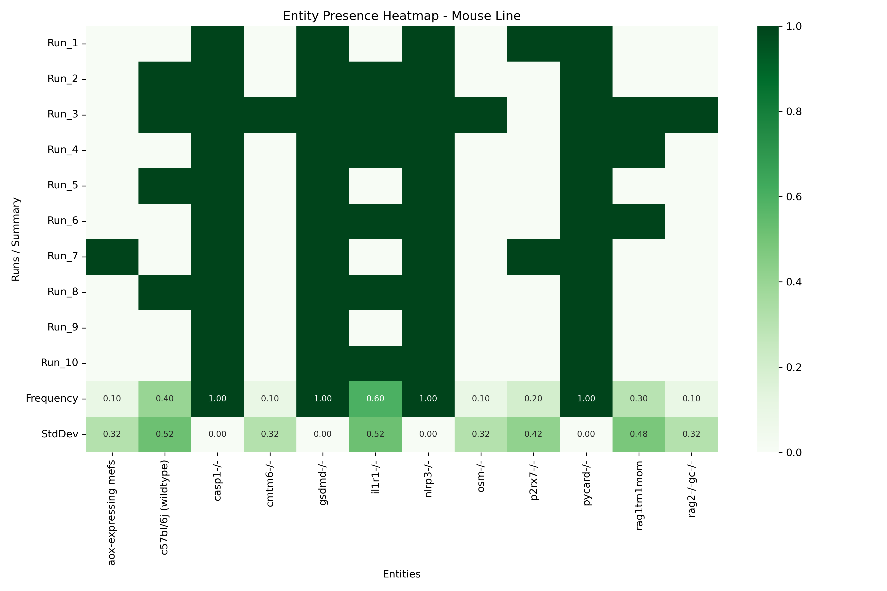
**

**
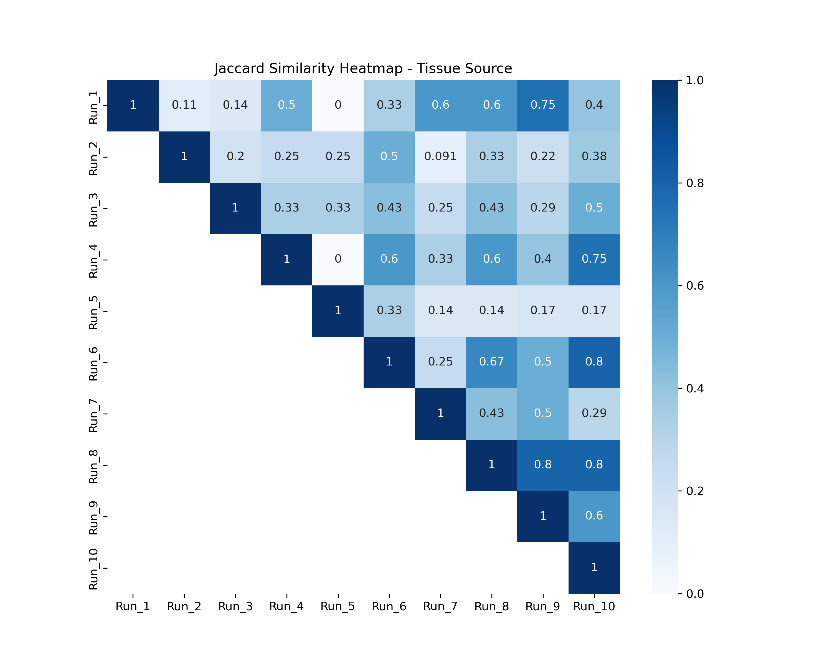

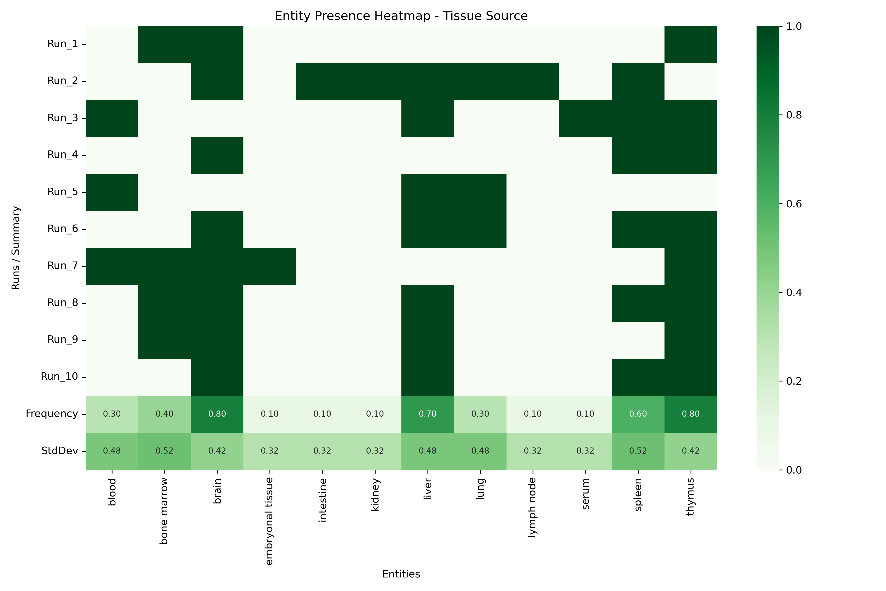
***Figure S9: Summary Jaccard heatmap (left) and the entities overlap table for the category Mouse Line (paper Saller et al 2025). The 4-step approach was run N=10 times on the plain paper.*

*Figure S10: Summary Jaccard heatmap (left) and the entities overlap table for the category Tissue Source (paper Saller et al 2025). The 4-step approach was run N=10 times on the plain paper.*

**
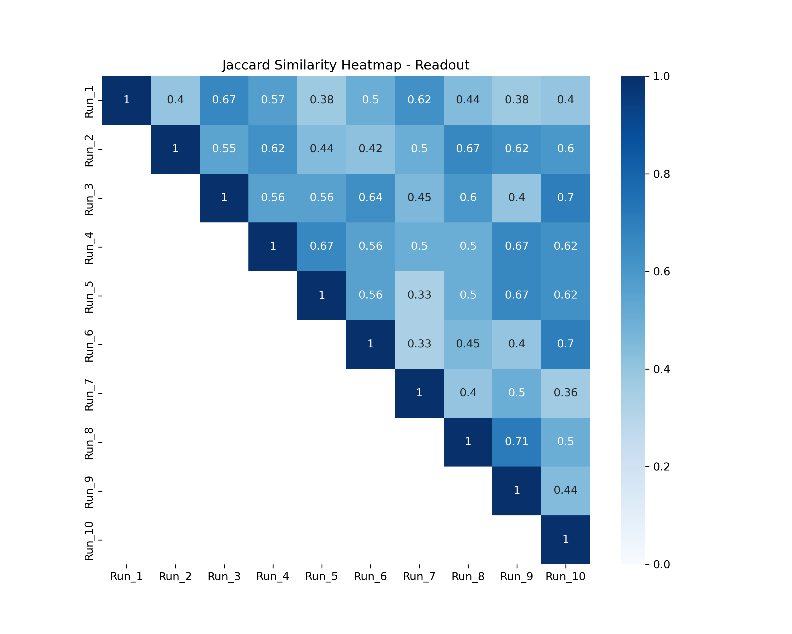

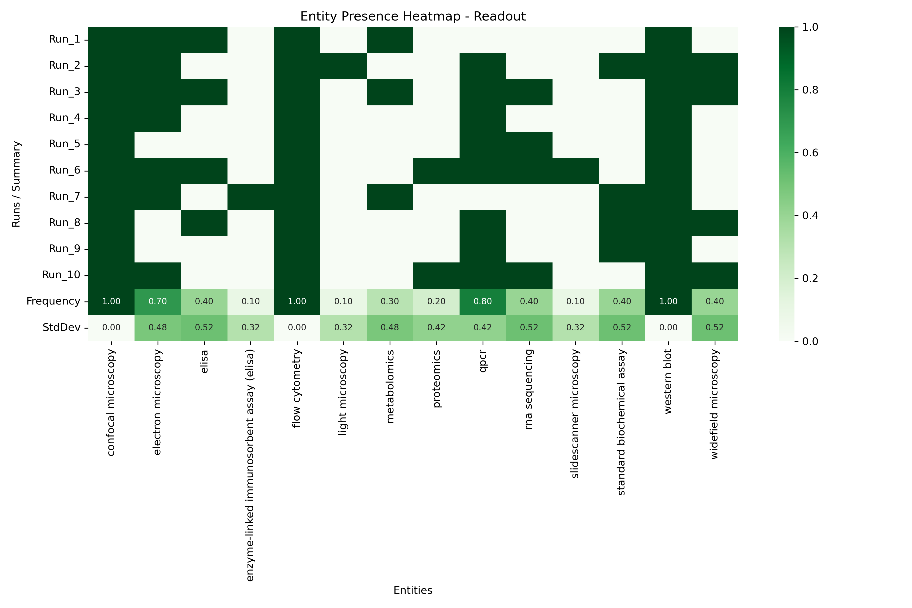
**

*Figure S11: Summary Jaccard heatmap (left) and the entities overlap table for the category Readout (paper Saller et al 2025). The 4-step approach was run N=10 times on the plain paper.*

**Wilcoxon Signed-Rank-Test Table**

*Table S4: Wilcoxon test results (p-value) for compatibility of number of suggested entries between paper without and with supplement (N=20), per category and overall number of suggestions (without Data availability).*

| **category** | **p-value** |
| --- | --- |
| CellLine | 0.794 |
| DataAvailability | 0.660 |
| HealthStatus | 0.414 |
| MouseLine | 0.951 |
| Oncogenes | 0.265 |
| Organism | 0.317 |
| PubTatorNotClassified | 0.706 |
| Readout | 0.365 |
| SamplePreparation | 0.750 |
| SampleProcessing | 0.222 |
| TissueSource | 1.000 |
| **Overall result** | **0.556** |

Reference list

1. Apostolova P, Kreutmair S, Toffalori C, et al. Phase II trial of hypomethylating agent combined with nivolumab for acute myeloid leukaemia relapse after allogeneic haematopoietic cell transplantation-Immune signature correlates with response. Br J Haematol. Oct 2023;203(2):264-281. [doi: 10.1111/bjh.19007] [Medline: 37539479]
2. Biavasco F, Ihorst G, Wäsch R, et al. Therapy response of glucocorticoid-refractory acute GVHD of the lower intestinal tract. Bone Marrow Transplant. Oct 2022;57(10):1500-1506. [doi: 10.1038/s41409-022-01741-3] [Medline: 35768570]
3. Chen YB, Mohty M, Zeiser R, et al. Vedolizumab for the prevention of intestinal acute GVHD after allogeneic hematopoietic stem cell transplantation: a randomized phase 3 trial. Nat Med. Aug 2024;30(8):2277-2287. [doi: 10.1038/s41591-024-03016-4] [Medline: 38844797]
4. Czech M, Schneider S, Peltokangas N, et al. Lipocalin-2 expression identifies an intestinal regulatory neutrophil population during acute graft-versus-host disease. Sci Transl Med. Feb 21, 2024;16(735):eadi1501. [doi: 10.1126/scitranslmed.adi1501] [Medline: 38381845]
5. de Maissin H, Groß PR, Mohiuddin O, et al. In Vivo metabolic imaging of [1-13 C]Pyruvate-d3 Hyperpolarized by reversible exchange with parahydrogen. Angew Chem Int Ed Engl. Sep 4, 2023;62(36):e202306654. [doi: 10.1002/anie.202306654] [Medline: 37439488]
6. Edwards-Hicks J, Apostolova P, Buescher JM, et al. Phosphoinositide acyl chain saturation drives CD8^+^ effector T cell signaling and function. Nat Immunol. Mar 2023;24(3):516-530. [doi: 10.1038/s41590-023-01419-y] [Medline: 36732424]
7. El Khawanky N, Hughes A, Yu W, et al. Demethylating therapy increases anti-CD123 CAR T cell cytotoxicity against acute myeloid leukemia. Nat Commun. Nov 8, 2021;12(1):6436. [doi: 10.1038/s41467-021-26683-0] [Medline: 34750374]
8. Frueh JT, Campe J, Sunaga-Franze DY, et al. Interferon regulatory factor 4 plays a pivotal role in the development of aGVHD-associated colitis. Oncoimmunology. 2024;13(1):2296712. [doi: 10.1080/2162402X.2023.2296712] [Medline: 38170159]
9. Grüninger PK, Uhl F, Herzog H, et al. Functional characterization of the PI3K/AKT/MTOR signaling pathway for targeted therapy in B-precursor acute lymphoblastic leukemia. Cancer Gene Ther. Nov 2022;29(11):1751-1760. [doi: 10.1038/s41417-022-00491-0] [Medline: 35794338]
10. Haring E, Andrieux G, Uhl FM, et al. Therapeutic targeting of endoplasmic reticulum stress in acute graft-versus-host disease. Haematologica. Jul 1, 2022;107(7):1538-1554. [doi: 10.3324/haematol.2021.278387] [Medline: 34407601]
11. Ho J, Schmidt D, Lowinus T, et al. Targeting MDM2 enhances antileukemia immunity after allogeneic transplantation via MHC-II and TRAIL-R1/2 upregulation. Blood. Sep 8, 2022;140(10):1167-1181. [doi: 10.1182/blood.2022016082] [Medline: 35853161]
12. Jaeger A, Gambheer SM, Sun X, et al. Activated granulocytes and inflammatory cytokine signaling drive T-cell lymphoma progression and disease symptoms. Blood. Jun 8, 2023;141(23):2824-2840. [doi: 10.1182/blood.2022015653] [Medline: 36696631]
13. Langenbach M, Giesler S, Richtsfeld S, et al. MDM2 inhibition enhances immune checkpoint inhibitor efficacy by increasing IL15 and MHC class II production. Mol Cancer Res. Aug 1, 2023;21(8):849-864. [doi: 10.1158/1541-7786.MCR-22-0898] [Medline: 37071397]
14. Maas-Bauer K, Stell AV, Yan KL, et al. ROCK1/2 signaling contributes to corticosteroid-refractory acute graft-versus-host disease. Nat Commun. Jan 10, 2024;15(1):446. [doi: 10.1038/s41467-024-44703-7] [Medline: 38199985]
15. Neuwirt E, Magnani G, Ćiković T, et al. Tyrosine kinase inhibitors can activate the NLRP3 inflammasome in myeloid cells through lysosomal damage and cell lysis. Sci Signal. Jan 17, 2023;16(768):eabh1083. [doi: 10.1126/scisignal.abh1083] [Medline: 36649377]
16. Saller BS, Wöhrle S, Fischer L, et al. Acute suppression of mitochondrial ATP production prevents apoptosis and provides an essential signal for NLRP3 inflammasome activation. Immunity. Jan 14, 2025;58(1):90-107. [doi: 10.1016/j.immuni.2024.10.012] [Medline: 39571574]
17. Schmidt D, Endres C, Hoefflin R, et al. Oncogenic calreticulin induces immune escape by stimulating TGFβ expression and regulatory T-cell expansion in the bone marrow microenvironment. Cancer Res. Sep 16, 2024;84(18):2985-3003. [doi: 10.1158/0008-5472.CAN-23-3553] [Medline: 38885318]
18. Socié G, Niederwieser D, von Bubnoff N, et al. Prognostic value of blood biomarkers in steroid-refractory or steroid-dependent acute graft-versus-host disease: a REACH2 analysis. Blood. Jun 1, 2023;141(22):2771-2779. [doi: 10.1182/blood.2022018579] [Medline: 36827620]
19. Talvard-Balland N, Braun LM, Dixon KO, et al. Oncogene-induced TIM-3 ligand expression dictates susceptibility to anti-TIM-3 therapy in mice. J Clin Invest. Jun 25, 2024;134(16):e177460. [doi: 10.1172/JCI177460] [Medline: 38916965]
20. Villa M, Sanin DE, Apostolova P, et al. Prostaglandin E_2_ controls the metabolic adaptation of T cells to the intestinal microenvironment. Nat Commun. Jan 11, 2024;15(1):451. [doi: 10.1038/s41467-024-44689-2] [Medline: 38200005]
21. Vinnakota JM, Biavasco F, Schwabenland M, et al. Targeting TGFβ-activated kinase-1 activation in microglia reduces CAR T immune effector cell-associated neurotoxicity syndrome. Nat Cancer. Aug 2024;5(8):1227-1249. [doi: 10.1038/s43018-024-00764-7] [Medline: 38741011]
22. Zeiser R, Lee SJ. Three US Food and Drug Administration-approved therapies for chronic GVHD. Blood. Mar 17, 2022;139(11):1642-1645. [doi: 10.1182/blood.2021014448] [Medline: 35081254]
23. Zeiser R, Socié G, Schroeder MA, et al. Efficacy and safety of itacitinib versus placebo in combination with corticosteroids for initial treatment of acute graft-versus-host disease (GRAVITAS-301): a randomised, multicentre, double-blind, phase 3 trial. Lancet Haematol. Jan 2022;9(1):e14-e25. [doi: 10.1016/S2352-3026(21)00367-7] [Medline: 34971577]
